# Ultrasound for Material Characterization and Processing

**DOI:** 10.3390/ma14143891

**Published:** 2021-07-12

**Authors:** Francesca Lionetto

**Affiliations:** Department of Engineering for Innovation, University of Salento, Via Monteroni, 73100 Lecce, Italy; francesca.lionetto@unisalento.it

Ultrasonic waves are nowadays used for multiple purposes in many different fields from the non-destructive inspection of materials to sonochemical synthesis of materials and welding. Usually, ultrasonic applications are divided into low intensity/high frequency ultrasound and high intensity/low frequency ultrasound. Low intensity ultrasound transmits energy through the medium in order to obtain the information about the medium or to convey information through the medium [1]. It is successfully used in non-destructive inspection, ultrasonic dynamic analysis, ultrasonic rheology, ultrasonic spectroscopy of materials, process monitoring, applications in civil engineering, aerospace and geological materials and structures and in the characterization of biological media [2]. Nowadays, it is an essential tool for assessing metals, plastics, aerospace composites, wood, concrete and cement [3]. High intensity ultrasound deliberately affects the propagation medium through the high local temperatures and pressures generated [4]. It is used in industrial processes such as welding, cleaning, emulsification, atomization, etc.; chemical reactions and reactor induced by ultrasonic waves; synthesis of organic and inorganic materials; microstructural effects; heat generation; accelerated material characterization by ultrasonic fatigue testing; food processing, environmental protection [5].

The Special Issue *Ultrasound for Material Characterization and Processing* collects eleven papers, one review and ten research papers, with the aim to present recent advances in ultrasonic wave propagation applied for the characterization or the processing of materials. Both fundamental science and applications of ultrasound in the field of material characterization and material processing have been gathered.

As regards low intensity ultrasound, Waldner et al. [6] in their review entitled “*Ultrasonic liquid penetration measurement in thin sheets—physical mechanisms and interpretation*”, give an overview on the three possible mechanisms for ultrasonic liquid penetration (ULP) measurements for the characterization of intrinsic properties of porous sheets. The review discusses the interpretation of ULP results, which are complex, as the ultrasound signal can be affected by several mechanisms. Then, it describes the individual physical mechanisms leading to different shapes of the ultrasound intensity curves, and provides measurements confirming that the measured ultrasound intensity is linked to the liquid penetration regime into the structure.

Acquaticci et al. [7] in the paper entitled “*Ultrasound Axicon: systematic approach to optimize focusing resolution through human skull bone*” propose a numerical approach for designing axicon lenses for many high-resolution applications, such as mapping or detection. The aim is to generate a focused ultrasound field by plane transducer using axicon lens, which can be suitable for focused ultrasound through human skull bone. The numerical simulations help to find the optimized parameters for the proposed system design. The sound field testing using hydrophone shows the performance of the proposed method by comparing the cases with and without a human skull phantom.

Lin et al. [8] in the paper entitled “*Effects of loading and boundary conditions on the performance of ultrasound compressional viscoelastography: a computational simulation study to guide experimental design*” present a finite element analysis of compressional viscoelastography. This ultrasonic imaging technique is used for measuring the viscoelastic properties of biomaterials and tissues and it is based on the analysis of the creep behavior of a material under an external mechanical compression. The authors demonstrate that loading conditions (the distribution of the applied compressional pressure on the surface of the sample) and boundary conditions (the fixation method used to stabilize the sample) can severely affect the measurement accuracy of the viscoelastic properties of materials.

Ryuzono et al. [9] in the paper entitled “*Topology optimization-based damage identification using visualized ultrasonic wave propagation*” present a new damage identification method in structures based on topology optimization in combination with visualized ultrasonic wave propagation. The proposed method is applied to an aluminum plate with an artificial crack and the estimated damage state and the sensitivity of the objective function were compared with numerical predictions.

Lionetto et al. [10] in the paper entitled “*Out-of-plane permeability evaluation of carbon fiber preforms by ultrasonic wave propagation*” present a novel experimental set-up, based on ultrasonic wave propagation, for the determination of the out-of-plane permeability of carbon fiber reinforcements, which is the dominant property in the infusion of large and flat panels with a high thickness. An accurate characterization of the reinforcement permeability is fundamental in order to estimate the optimum process parameters for manufacturing high-quality components. The experimental results, obtained in unsteady conditions, have been compared with those obtained in steady conditions by a traditional gravimetric method and validated by some analytical models. The work demonstrates the feasibility and potential of the proposed set-up for permeability measurements thanks to its non-invasive character and the one-side access.

In the area of structural mechanics, concrete structures and nondestructive testing Zima et al. [11] in the paper entitled “*Numerical study of concrete mesostructure effect on lamb wave propagation*” analyze the propagation of guided wave in concrete plates accounting for the heterogeneous structure of concrete. The influence of aggregate ratio and particle size on dispersion curves representing Lamb wave modes is analyzed by several concrete models with randomly distributed aggregates, generated by means of the Monte Carlo method. The study shows that Lamb wave propagates with different velocities in homogeneous and heterogeneous models and the difference increases with aggregate ratio and particle size. This result demonstrates the potential of the method for the monitoring of concrete structures.

Wang et al. [12] in the paper entitled “*Monitoring the Setting Process of Cementitious Materials Using Guided Waves in Thin Rods*” demonstrate the reliability of an ultrasonic method based on embedded guided waves to monitor the early-age properties of mortar and concrete samples through continuous attenuation monitoring. The technique is able to eliminate the effects of coarse aggregates, which makes it of great potential to be directly applied in situ to the fresh concrete. The method has the potential to be used when additives are added to concrete, thus changing its rheological properties.

Concerning the applications of high intensity ultrasound, Frederick et al. [13], in the paper entitled “*Disassembly study of ultrasonically welded thermoplastic composite joints via resistance heating*”, demonstrate a novel concept in the disassembly of ultrasonically welded thermoplastic composite joints. The recycling of thermoplastic composite joints is realized by resistance heating via a nanocomposite film located at the welded interface. This electrically conductive film, containing multi-walled carbon nanotubes, is able to promote joint disassembly through a combination of nanocomposite and matrix melting and weakened fiber–matrix interface.

Shan et al. [14], in the paper entitled “*Building of longitudinal ultrasonic assisted turning system and its cutting simulation study on bulk metallic glass*”, present an experimental-numerical study on a novel machining technology, longitudinal ultrasonic assisted turning. After presenting this technology, they establish a two-dimensional finite element model of the ultrasonic assisted turning, aimed at studying the effect of ultrasonic vibration on cutting force under different cutting speeds on bulk metallic glasses. The latter are a new kind of material, which are made by rapid condensation of alloy with excellent properties such as high strength, high hardness and corrosion resistance, but they are hard to be machined. The results demonstrate that longitudinal ultrasonic vibration can significantly reduce the average cutting force as well as the von Mises stress when the turning speed is below the critical turning speed.

Shi et al. [15], in the paper entitled “*Effects of ultrasonic bending vibration introduced by an L-shaped ultrasonic rod on the microstructure and properties of a 1060 aluminum alloy strip formed by twin-roll casting*”, present a new type of ultrasonic generator to assist the process of continuous casting and rolling of aluminum alloy. A novel L-shaped ultrasonic rod is designed to introduce an ultrasonic bending vibration into the aluminum melt. The ultrasonic bending vibration improves the microstructure of the 1060 aluminum alloy cast rolling strip, characterized by grain refinement, and consequently its properties.

Shi et al. [16], in the paper entitled “*Effect of ultrasonic bending vibration introduced by the L-shaped ultrasonic rod on solidification structure and segregation of large 2A14 ingots*”, propose a new design of L- shaped ceramic ultrasonic wave guide rod for the introduction of ultrasonic bending vibration during the solidification of the 2A14 aluminum alloy. The authors demonstrate the advantages in terms of grain refinement, deagglomeration of the secondary phase, and improving the ingot macrostructure.

## References

[1] Lionetto F., Maffezzoli A., Ottenhof M.A., Farhat I.A., Mitchell J.R. (2006). Ultrasonic investigation of wheat starch retrogradation. J. Food Eng..

[2] Espinoza-Gonzalez C., Avila-Orta C., Martinez-Colunga G., Lionetto F., Maffezzoli A. (2016). A Measure of CNTs Dispersion in Polymers with Branched Molecular Architectures by UDMA. IEEE Trans. Nanotechnol..

[3] Lionetto F., Maffezzoli A. (2005). Relaxations during the postcure of unsaturated polyester networks by ultrasonic wave propagation, dynamic mechanical analysis, and dielectric analysis. J. Polym. Sci. Part B Polym. Phys..

[4] Lionetto F., López-Muñoz R., Espinoza-González C., Mis-Fernández R., Rodríguez-Fernández O., Maffezzoli A. (2019). A Study on Exfoliation of Expanded Graphite Stacks in Candelilla Wax. Materials.

[5] Dell’Anna R., Lionetto F., Montagna F., Maffezzoli A. (2018). Lay-Up and Consolidation of a Composite Pipe by In Situ Ultrasonic Welding of a Thermoplastic Matrix Composite Tape. Materials.

[6] Waldner C., Hirn U. (2020). Ultrasonic liquid penetration measurement in thin sheets—Physical mechanisms and interpretation. Materials.

[7] Acquaticci F., Lew S.E., Gwirc S.N. (2019). Ultrasound axicon: Systematic approach to optimize focusing resolution through human skull bone. Materials.

[8] Lin C.-Y., Chang K.-V. (2021). Effects of Loading and Boundary Conditions on the Performance of Ultrasound Compressional Viscoelastography: A Computational Simulation Study to Guide Experimental Design. Materials.

[9] Ryuzono K., Yashiro S., Nagai H., Toyama N. (2020). Topology optimization-based damage identification using visualized ultrasonic wave propagation. Materials.

[10] Lionetto F., Montagna F., Maffezzoli A. (2020). Out-Of-Plane Permeability Evaluation of Carbon Fiber Preforms by Ultrasonic Wave Propagation. Materials.

[11] Zima B., Rafael K. (2020). Numerical Study of Concrete Mesostructure Effect on Lamb Wave Propagation. Materials.

[12] Wang D., Yu G., Liu S., Sheng P. (2021). Monitoring the Setting Process of Cementitious Materials Using Guided Waves in Thin Rods. Materials.

[13] Frederick H., Li W., Palardy G. (2021). Disassembly Study of Ultrasonically Welded Thermoplastic Composite Joints via Resistance Heating. Materials.

[14] Shan S., Feng P., Zha H., Feng F. (2020). Building of Longitudinal Ultrasonic Assisted Turning System and Its Cutting Simulation Study on Bulk Metallic Glass. Materials.

[15] Shi C., Fan G., Mao X., Mao D. (2020). Effects of ultrasonic bending vibration introduced by an L-shaped ultrasonic rod on the microstructure and properties of a 1060 aluminum alloy strip formed by twin-roll casting. Materials.

[16] Shi C., Wu Y., Mao D., Fan G. (2020). Effect of Ultrasonic Bending Vibration Introduced by the L-shaped Ultrasonic Rod on Solidification Structure and Segregation of Large 2A14 Ingots. Materials.

